# Measuring Activated Clotting Time during Cardiac Catheterization and PCI: The Effect of the Sampling Site

**DOI:** 10.1155/2021/4091289

**Published:** 2021-09-11

**Authors:** J. C Heemelaar, T. Berkhout, A. A. C. M. Heestermans, J. C. Zant, A. M. J. de Vos, N. J. W. Verouden, M. T. Dirksen, J. van Ramshorst

**Affiliations:** ^1^Department of Cardiology, Northwest Clinics, Alkmaar, Netherlands; ^2^Department of Clinical Chemistry, Northwest Clinics, Alkmaar, Netherlands; ^3^Department of Cardiology, Catharina Hospital, Eindhoven, Netherlands; ^4^Department of Cardiology, Amsterdam University Medical Center, Amsterdam, Netherlands

## Abstract

**Results:**

In 100 patients (mean age 67.1, 65% male), no significant differences were observed in ACT values obtained from the guiding catheter and arterial sheath (mean difference (MD) −18.3 s; standard deviation (SD) 96 s; *P*=0.067). Contrarily, ACT values obtained from the intravenous line were significantly lower as compared to values obtained from the guiding catheter (MD 25.7 s; SD 75.5; *P*=0.003) and arterial sheath (MD 39 s; SD 102.8; *P* < 0.001). Furthermore, ACT measurements from the arterial sheath showed a statistically significant proportional bias when compared to the other sampling sites (sheath vs. catheter, *r* = 0.761, *P*=0.001; sheath vs. IVL, *r* = 1.013, *P* < 0.001).

**Conclusions:**

The present study shows statistical significance and possibly clinically relevant variations between ACT measurements from different sample sites. Bias in ACT measurements may be minimized by using uniform protocols for ACT measurement during cardiac catheterization.

## 1. Introduction

Unfractionated heparin (UFH) is the mainstay of antithrombotic therapy for the prevention of thrombus formation in coronary angiography (CAG) and percutaneous coronary intervention (PCI). Since the mid-1970s, UFH has been used to prevent thrombus formation in patients during bypass surgery and soon became common practice for patients undergoing PCI for the reduction of ischemic complications [1–6]. The inconsistency of dose and effect of UFH led to the development of new direct thrombin inhibitors such as bivalirudin to provide anticoagulation, for instance, during PCI in patients with acute myocardial infarction [7, 8]. Prevention of stent thrombosis nowadays consists of both antiplatelet and antithrombotic agents [9, 10]. Bleeding complications have been the main adverse event of anticoagulant therapy and are a critical determinant of fatal and nonfatal outcomes after primary PCI in acute myocardial infarction [11, 12]. Finding a balance between the reduction of ischemic events and bleeding complications is paramount to optimizing the outcomes of coronary interventions. In the rapidly evolving field of interventional cardiology, the use of state-of-the-art intracoronary imaging modalities and microcatheters has led to treatment of more complex lesions. These procedures beget longer operating times, and maintaining adequate anticoagulation becomes increasingly more important as it may influence the incidence of ischemic and bleeding complications during these complex procedures.

The activated clotting time (ACT) reflects UFH activity and has been used for decades to monitor heparin dosage [1]. However, standardized protocols or clinical guidelines for the use of ACT measurement in cardiac catheterization are lacking. At this time, it is unknown if the site of blood sampling influences ACT values. For instance, blood could be obtained from arterial as well as venous blood samples. Theoretically, sampling blood from heparin-coated access sites (arterial sheath and diagnostic or guiding catheter) might influence ACT values and result in an incorrect representation of coagulation status.

To our best knowledge, no studies aiming at standardisation of ACT measurements at the catheterization laboratory have been currently conducted. Therefore, the aim of the present study is to investigate the influence of the sampling site on the variability of ACT values at the end of coronary angiography or PCI.

## 2. Materials and Methods

### 2.1. Study Design

We conducted a cross-sectional, single-center, observational method comparison study that compared the activated clotting time (ACT) after elective cardiac catheterization with preprocedural unfractionated heparin (UFH) administration, derived from three different sample sites: the arterial catheter, arterial sheath, and peripheral intravenous line (IVL). The study was designed and performed by the Department of Cardiology at Noordwest Ziekenhuisgroep in Alkmaar, the Netherlands.

### 2.2. Study Population

Consecutive patients scheduled for diagnostic coronary angiography (CAG) or elective percutaneous coronary intervention (PCI) were screened for entry into this study. Patients were eligible for inclusion when it was expected that the scheduled procedure would be accomplished with a single bolus of heparin. Practically, this implied that the expected duration of the procedure would not exceed 1 hour.

The exclusion criteria were concurrent medication use or conditions that would interfere with ACT-measurements, i.e., use of novel oral anticoagulants or vitamin K antagonist, liver function disorders with coagulopathies (defined as PT-INR greater than 2.0 or thrombocyte count <100 × 10^9^), chronic use of nonsteroid anti-inflammatory drugs (with the exception of aspirin), and known renal insufficiency (e.g., a serum creatinine level of greater than 265 *μ*mol/L (3.5 mg/L). Furthermore, patients with unstable coronary artery disease or hemodynamical instability at the time of the procedure, inability to comprehend the Dutch language, or previous participation in this study were excluded.

When patients fulfilled the abovementioned selection criteria, they were asked for written informed consent. The study was conducted according to the principles of the Declaration of Helsinki, the Medical Research Involving Human Subjects Act (WMO), and the Good Clinical Practice guidelines and with the approval of the local medical ethical committee.

### 2.3. Protocol and Materials

After written consent was given, each patient was asked for cardiac complaints, risk factors for coronary artery disease, and coagulopathies at the time of inclusion. Current medication use was registered. A physical examination was performed including vital functions, functional status according to the Canadian Cardiovascular Society (CCS), and New York Heart Association (NYHA) classification.

All cardiac catheterizations were performed according to the local protocols. The arterial sheath and arterial catheters were flushed with heparinized saline before introduction. After radial sheath insertion (Glidesheath Slender®, Terumo Europe, Leuven, Belgium) in diagnostic coronary angiography, a bolus of 5000 international units (IU) of UFH was administered through the arterial sheath and subsequently flushed with saline. Patients who underwent elective PCI received 70–100 IU/kg at the discretion of the interventionalist. At the end of the procedure, before arterial sheath removal, blood was simultaneously obtained from a peripheral IVL from the arm, from the catheter in the ascending aorta, and from the arterial sheath.

Blood samples were immediately analysed with the i-Stat® device (ACT-k cartridge, Abbott, Princeton, NJ, United States of America) in the catheterization lab. ACT values and timing of blood sampling are recorded on paper by the research nurse or catheterization lab assistant. All cartridges were prewarmed according to the manufacturer's instructions. The i-Stat® device was maintained according to the manufacturer's quality assurance to verify proper instrument performance.

If the maximum value of measurable ACT was derived from a blood sample (1000 seconds), it was considered a false measurement and the measurement was excluded from the analysis. All measurements below this maximum level were accepted.

### 2.4. Statistical Analysis

All patients from whom blood samples were successfully obtained from more than one sampling site were included in the analysis. A two-tailed *t*-test was used to compare the systematic difference between two sample sites. A two-sided alpha level of 0.05 was used to test for statistical significance.

To visualise agreement between sampling sites, Bland–Altman plots were constructed for the three comparisons of measurement methods, i.e., catheter versus arterial sheath, catheter versus IVL, and arterial sheath versus IVL. The systematic difference between the two measurement methods was tested for statistical significance. The 95% limits of agreement were defined as systematic difference ±1.96 SD. Linear regression was used to assess proportional bias between the two methods, i.e., when methods do not agree equally through the range of measurements.

Extreme values were defined as mean ACT values greater than the 95% limits of agreement.

## 3. Results

**Figure 1:**
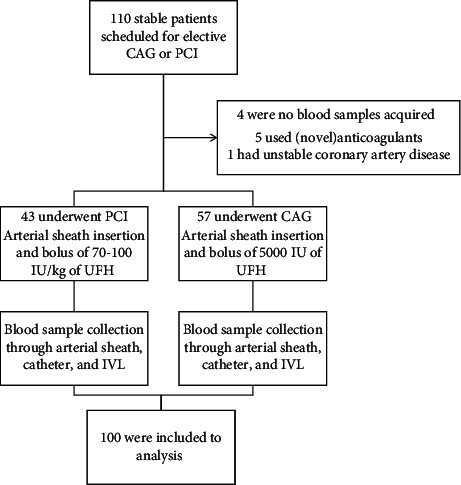
Screening, procedure and analysis^*∗*^. CAG = coronary angiography; PCI = percutaneous intervention; IU = International Units; UFH = unfractionated heparin; IVL = intravenous line; ^*∗*^patients were eligible for inclusion when it was expected that the scheduled procedure would be accomplished with a single bolus of heparin. At the start of the procedures, an injection of UFH was administered over the arterial sheath. Patients who underwent CAG or PCI received, respectively, 5000 IU or 70–100 IU/kg of UFH. At the end of the procedure, blood samples from the arterial sheath, catheter, and IVL were collected and the ACT measurement was performed.

In a four-month period, 110 subsequent patients met the inclusion criteria and gave written informed consent (Figure 1). A group of nine patients were excluded from analysis due to novel anticoagulant or vitamin K antagonist use (*N* = 5) or failure to obtain blood samples from two or more sampling sites (*N* = 4). One patient was excluded due to unstable coronary artery disease at time of cardiac catheterization. As a result, 100 patients were included in analysis.

Thus, 100 patients (mean age 67.1 y, SD 11.3, 65% males) were included in the present study. Baseline characteristics are summarized in Table 1. In brief, 57 patients underwent diagnostic CAG and 43 patients underwent elective PCI. The majority of patients (90%) were on antiplatelet therapy, of whom 47% were on dual antiplatelet therapy; in 3 cases, a second bolus of heparin was administrated during the procedure on the operators' discretion.

Successful ACT measurements were performed in the arterial catheter, arterial sheath, and IVL in 96, 97, and 86 cases, respectively. Subsequently, 94 comparisons could be made between arterial catheter and sheath measurements, whereas IVL measurements could be compared in 82 cases with arterial catheter measurements and in 83 cases with arterial sheath measurements.

**Figure 2:**
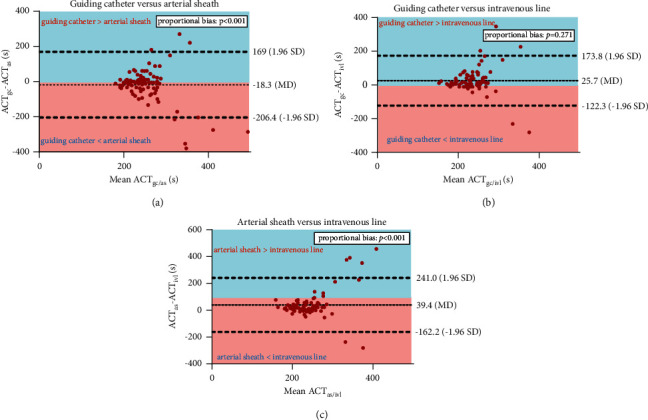
Bland–Altman plots of three ACT sample site comparisons. ACT = activated clotting time; AS = arterial sheath; GC = guiding catheter; IVL = peripheral intravenous line; MD = mean difference; and SD = standard deviation. Systematic differences in ACT between the (a) guiding catheter and arterial sheath, (b) guiding catheter and intravenous line, and (c) arterial sheath and intravenous line.

The mean ACT value of catheter, sheath, and IVL measurements was, respectively, 244 s (SD 53), 262 s (SD 80), and 221 s (SD 54) (Table 2). There was no significant difference in mean ACT value between guiding catheter and arterial sheath measurements (mean difference (MD), −18.3 s; SD, 96 s; *P*=0.067). The mean ACT value in IVL measurements was significantly lower compared to guiding catheter measurements (MD, 25.7 s; SD, 75.5; *P*=0.003) and arterial sheath measurements (MD, 39 s; SD 102.8, *P* < 0.001). A Bland–Altman plot was constructed for all sampling site comparisons (Figure 2). These plots revealed a statistically significant proportional bias between arterial sheath measurements as compared with the other sampling sites (sheath vs. catheter, *r* = 0.761, *P*=0.001; sheath vs. IVL, *r* = 1.013, *P* < 0.001), implying larger random bias at higher ACT values when the arterial sheath is involved. No proportional bias was observed between catheter and IVL measurements (*r* = 0.221, *P*=0.271).

## 4. Discussion

The present study investigated the effect of the sampling site on ACT measurement in patients scheduled for diagnostic CAG or elective PCI. The present study allows characterizing the potential measurement variations between these sampling sites, although it was not designed to assess the clinical effects of any potential differences in ACT. The most important finding of the current study is that the sampling site substantially impacts the measured ACT value.

First, the current study showed that ACT values derived from the peripheral intravenous line are significantly lower than both arterial sheath- and catheter-derived measurements, indicating a structural difference between the peripheral IVL and the arterial measurements. This is in line with observations from a recent comparative study in patients undergoing cardiac surgery, which demonstrated prolonged clotting times in arterial blood as compared to venous samples [13]. The mechanism underlying these findings is currently unknown. It has been suggested that oxygen content influences coagulation by changing viscosity [14]. Furthermore, it has been suggested that shear forces at the sampling site stimulate coagulation, possibly by platelet activation [15]. Interestingly, since shear forces were the highest in the venous samples in this study (peripheral IVL), the findings of this study are consistent with this hypothesis.

Second, a proportional bias was observed between arterial sheath measurements and both arterial catheter and peripheral IVL, suggesting that the arterial sheath measurement of ACT is subject to larger deviations at higher values. One of the most important factors which may account for this finding is the presence of heparin in the lumen of the sheath and catheter earlier in the procedure. By protocol, both the sheath and catheter are prepared with heparinized saline before introduction. In addition, a bolus of heparin is administrated through the arterial sheath just after insertion. Thus, it may be hypothesized that small residues of heparin in the catheter and sheath may influence ACT values in blood withdrawn through the lumen of this sheath or catheter. Since heparin was administrated through the arterial sheath in all patients and only sporadically through the intravenous line, this may account for the relatively high number of deviations in the arterial sheath measurements.

To our knowledge, this is the first prospective study comparing ACT measurements from different sampling sites in a cardiac catheterization laboratory. Although the present study is obviously not designed to evaluate clinical endpoints resulting from ACT measurements, it can be appreciated that the observed variations between sampling sites can be relevant in the clinical setting. For example, according to the 2014 AHA guidelines on acute coronary syndromes, a periprocedural ACT value of less than 250 s justifies a second bolus of heparin because retrospective data showed an increase of major in-hospital ischemic events in lower ranges of ACT [3, 16]. In the current era of complex PCI and especially PCI of chronic total occlusions, ACT has emerged as an important tool to monitor coagulation during longer and complex procedures. During such procedures, ACT is frequently measured and higher but strictly defined cutoff values, such as 300 or 350 s, are used. Thus, even small variations can influence clinical decisions and outcome. On the other hand, it should be appreciated that the observed variations can be easily minimized by using a standardized protocol for ACT measurements demanding consistent use of one sampling site through which no heparin had been administrated (usually the guiding catheter). Based on the current study, an arterial sampling site may be preferred for this purpose.

### 4.1. Limitations

There are several limitations to take into account. As mentioned above, this is a method comparison study performed in the absence of a golden standard. Second, the patient cohort is relatively small. Especially due to the relatively low success rate of peripheral IVL-derived ACT measurements, less comparisons could be made with this method. Third, while the measurements were comparable in the lower ranges, in the higher ranges of ACT, the study is unpowered to make plausible conclusions on the comparability of the two sampling methods. Finally, all ACT measurements were performed with the i-Stat device, which has good correlation with the Hemochron ACT device but overall underestimates ACT as compared with values from the Hemochron device [17].

## 5. Conclusions

The results of the present study demonstrate that the site of blood sampling is an important factor regarding ACT measurement during cardiac catheterization. The observed variations in ACT measurements may be minimized by using uniform protocols for ACT measurement during cardiac catheterization. The results of the present study may contribute to the design of future studies investigating the clinical effects of ACT-guided anticoagulation during endovascular procedures.

## Figures and Tables

**Table 1:** Baseline characteristics of the study population.

	*N* = 100^*∗*^
Age, mean ± SD (yr)	67.1 ± 11.3
Male sex	65
Current smoker	26
Family history of cardiovascular disease	52
Arterial hypertension	60
Hypercholesterolaemia	49
Diabetes- insulin dependent	11
Diabetes- noninsulin dependent	12
Peripheral artery disease	3

Medication use at admission	
Antiplatelet therapy	90
Dual antiplatelet therapy	47
ACE inhibitors or angiotensin-receptor blockers	43
Beta-blockers	80
Calcium channel blockers	27
Statins	82

Cardiac symptoms	
Angina	81
Dyspnoea	17
Palpitations	7

Procedure	
Coronary angiography	57
Percutaneous coronary intervention	43

^*∗*^Exactly 100 patients were included in analysis. Therefore, for the convenience of the reader, only absolute count is noted, as it is the same as the percentage. ACE = angiotensin-converting enzyme; SD = standard deviation.

**Table 2:** Sample site comparison^*∗*^.

Sampling site	Successful measurements	Mean ACT (s)	SD (s)
Catheter	96	244	53
Sheath	97	262	80
IVL	86	221	54

Matched comparisons	ACT mean difference (s)	SD	*P* value
Catheter vs. sheath (*N* = 94)	−18.3	96.0	0.067
Catheter vs. IVL (*N* = 82)	25.7	75.5	0.003^*∗∗*^
Sheath vs. IVL (*N* = 83)	39.0	102.8	0.001^*∗∗*^

^*∗*^ACT values obtained had a significantly lower ACT values as compared to both arterial catheter and arterial sheath. There were no significant differences between ACT values from arterial catheter and arterial sheath blood samples. ^*∗∗*^Statistically significant difference in the two-tailed paired T-test (*α* = 0.05). s = seconds; SD = standard deviation; IVL = intravenous line.

## Data Availability

The data used to support the findings of this study are included within the article.

## Abbreviations

ACT: Activated clotting time
AHA: American Heart Association
CCS: Canadian Cardiovascular Society
IVL: Intravenous line
NYHA: New York Heart Association
UFH: Unfractionated heparin.
